# Scaling up availability, accessibility, quality and equity – highlights from the 4th Uganda conference on cancer and palliative care, held in Kampala, Uganda

**DOI:** 10.3332/ecancer.2023.1628

**Published:** 2023-11-13

**Authors:** Julia Downing, Nixon Niyonzima, Eddie Mwebesa, Lisa Christine Irumba, Judith Asasira, Bernadette Basemera, Diana Basirika, Alfred Jatho, Immaculate Mbarusha, Harriet Nalubega, Dorothy Olet Adong, Deirdre Ryan, Danait Tesfai, Cynthia Kabagambe, Joyce Zalwango, Catherine Amuge, Cissy Nassolo, Edward Kakungulu, Jackson Orem, Mark Mwesiga

**Affiliations:** 1 International Children’s Palliative Care Network, Suite 1b, Whitefrairs, Lewins Mead, BS1 2NT Bristol, UK; 2 Palliative Care Education and Research Consortium, PO Box 6245, Kyadondo Block 262, Plot 9, Kibuye, Makindye, Kampala, Uganda; 3 Makerere University, University Road, Kampala, Uganda; 4 Uganda Cancer Institute, Upper Mulago Hill Road, Kampala, Uganda; 5 Hospice Africa Uganda, Mobutu Road, Kampala, Uganda; 6 Palliative Care Association of Uganda, Block 383, Plot 8804, Kitende, Entebbe Road, Busiro, Uganda; 7 Clark International University, Kawagga Close, Off Kalungi Road, Muyenga Block 244, Kampala, Uganda

**Keywords:** cancer care, palliative care, Uganda, innovation, policy, integration, education, research, paediatrics, quality

## Abstract

The 4th Uganda Conference on Cancer and Palliative Care was held from the 14^th^–15^th^ September 2023. It was run jointly by the Uganda Cancer Institute and the Palliative Care Association of Uganda, in collaboration with the Ministry of Health. The conference was held at the Speke Resort, Munyonyo and 450 participants came together for a face-to-face conference following the virtual one held in 2021. It was an opportunity for all those working in the fields of cancer and palliative care to come together, to share lessons and learn from each other, as well as celebrate 30 years since specialist palliative care came to Uganda. The conference was officially opened by the Commissioner for Non-Communicable Diseases on behalf of the Minister of Health, who reiterated the Government’s commitment to reducing the burden of cancer and expanding the provision of palliative care within Uganda. Dr Tedros Adhanom Ghebresus, the Director General of the World Health Organization welcomed participants to the conference, and the Assistant Bishop of Kampala Diocese, the Right Reverend Hannington Mutebi shared his experience of living with cancer. The conference was organised into six tracks:* Innovations and new technologies; Education, advocacy, policy and law; Health promotion, prevention and early detection; Family and community involvement and empowerment; Clinical care and symptom management; and, Psychological, social and spiritual care*. The themes of paediatrics, vulnerable populations, service development and research were integrated throughout the tracks, and workshops were held that explored topics such as governance, access to essential medicines, national data reporting, research and education, and aging and ageism. Throughout the conference there was a sense of optimism, of resilience and a commitment to the ongoing development of cancer and palliative care services within the country.

## Introduction

The need for cancer and palliative care services is on the rise both globally, in East Africa and in Uganda. Since the 3rd Uganda Conference in 2021 [1] COVID restrictions on meetings have been lifted, thus this 4th Uganda Conference on Cancer and Palliative Care run jointly by the Uganda Cancer Institute (UCI) and the Palliative Care Association of Uganda (PCAU), in collaboration with the Ministry of Health (MoH), was held in-person at the Speke Resort, Munyonyo. Delegates had the opportunity to come together again after the pandemic and discuss issues around scaling up availability, accessibility, quality and equity in cancer and palliative care provision, applying lessons learnt throughout the pandemic. At the heart of this joint UCI-PCAU conference was the commitment to not only continue to provide and develop both cancer and palliative care services within the country, but also how these can be scaled up to reach more people and their families, whilst maintaining quality, and ensuring equity of services. The Honorable Dr Aceng Jane Ruth Ocero, Minister of Health reaffirmed ‘*our government’s unwavering commitment, as well as that of my Ministry, to expanding cancer and palliative care services in alignment with Universal Health Coverage principles. Our Ministry fully embraces the conference theme, ‘Scaling up availability, accessibility, quality and equity’’.* The Honourable Minister wished everyone a successful and enriching conference [2].

The conference was held on the 14th and 15th of September 2023, 30 years after the first specialist palliative care service was started in Uganda – an anniversary that was celebrated in a variety of ways throughout the conference, with several of those involved from the start in attendance. The PCAU is the National Association for palliative care providers in Uganda and ‘works in partnership with the MoH, other government ministries, agencies, departments, civil society and individuals to accelerate the integration of palliative care into the health care system in Uganda’ [2]. Founded in 1999, this year PCAU is celebrating 24 years of co-ordinating and advocating for palliative care in Uganda, with 25 Member Organisations and over 1,300 individuals, including 32 life members. One hundred and seven of the Districts in Uganda are now reached by palliative care, and there are 226 accredited palliative care services in the country [3].

The UCI was founded in 1967 through the Makerere Department of Surgery, the America National Cancer Institute and the British Empire Cancer Campaign and this year celebrated 56 years of service delivery. Initially, it was set up to facilitate clinical trials in cancers that were highly prevalent in East Africa and it is now the leading provider of cancer treatment and research in Uganda, providing comprehensive and state-of-the-art cancer services, promoting early detection, and improving outcomes for individuals affected by cancer. As the national cancer centre, UCI is ‘*committed to extending services to the masses through our strategy of setting up Regional Cancer centers in the Northern (Gulu), West Nile (Arua), Eastern (Mbale) and Western (Mbarara) regions of Uganda’* with limited services already being provided in Mbarara and Gulu [4].

Cancer is one of the leading causes of morbidity globally, the leading cause of death in low-and-middle-income countries and second only to cardiovascular disease in high income countries and is therefore an important public health concern [5]. In 2022 there was an estimated 19.3 million new cases and almost 10 million deaths [6, 7]. Globally rates are expected to increase to 28.4 million by 2040 [7]. In Uganda, where there is a population of 45.7 million people, there were an estimated 34,008 new cases with 22,992 deaths from cancer, with 62,548 5-year prevalent cases in 2020. The estimated risk of developing cancer before the age of 75 years was 15.8% and dying from cancer before the age of 75 years was 12%. The five most frequent cancers were Cervix uteri, Kaposi sarcoma, Breast, prostate and Non-Hodgkin Lymphoma, however it is estimated that only 20% of those in Uganda diagnosed with cancer access services [8]. In children it is estimated that 2,000 children in Uganda develop cancer and over 2,250 children die of cancer annually, however, in 2022 it was estimated that only 25% access care, although clear national data on childhood cancer is not available. The most common cancers among children in Uganda are leukaemia, lymphoma, nephroblastoma and rhabdomyosarcoma [9].

The Global Atlas of Palliative Care estimates that there are over 56.8 million people globally that need access to palliative care, with 25.7 million of those being in the last year of life [10]. However, only around 12% of these can access care. A study looking at children needing palliative care identified 21.1 million children globally needing palliative care with around 8 million needing access to specialist care [11], with only around 5%–10% of children and their families able to access care [12]. However, Uganda is seen as one of the leading countries in sub-Saharan Africa for the provision of palliative care [13], and both cancer and palliative care have been highlighted as a need through the World Health Assembly (WHA) passing resolutions which are critical in guiding us into the future for cancer [14] and palliative care service provision [15].

## Opening of the conference

The opening of the conference took place on the morning of the 14th September and commenced with thanksgiving and prayer by Rev Diana Nkesiga which was followed by a short video on the status of cancer and palliative care in Uganda. Participants were then welcomed by the conference chairs – Dr. Nixon Niyonzima (UCI) and Dr. Eddie Mwebesa (PCAU). They thanked the conference organising team along with the funders and wished all the delegates a wonderful conference, noting how pleased they were that we were once again able to meet face-to-face. The Assistant Bishop of Kampala Diocese, the Right Reverend Hannington Mutebi then shared his experience of living with leukaemia and undergoing a bone marrow transplant in the UK. He shared how when he had been told he had cancer he immediately thought of death, but that the health professionals caring for him had supported him and encouraged him – recognising the ‘one can be healed with effective communication and a word of encouragement’.

It was then the turn of the Executive Directors from PCAU and UCI to welcome everyone and they encouraged everyone to collaborate, to learn from each other and to celebrate our accomplishments in Uganda – particularly as we celebrate 30 years since the inception of palliative care in Uganda. However, they reminded participants that as we celebrate achievements, we must remember that the need for quality cancer and palliative care continues to rise and we must continue to move forward, learn and not be complacent. Dr Tedros Adhanom Ghebresus, the Director General of the World Health Organization also welcomed participants to the conference through a pre-recorded speech. He recognised the theme of the conference – ‘Scaling up availability, accessibility, quality and equity – stressing its importance as the prevalence of cancer is rising as is the need for palliative care services. He spoke of the WHO resources available to support scaling up of services, noting that the WHO is committed to scaling up both cancer and palliative care services to ensure that no one is left behind. He also thanked Dr Anne Merriman and all working in palliative care in Uganda for their work over the previous 30 years. He finished by saying that ‘*Together, we can support cancer and help patients live and die with dignity’*. The celebration of 30 years of palliative care in Uganda then continued as Rose Kiwanuka, Uganda’s first palliative care nurse and previous Country Director of PCAU shared the journey towards reducing health-related suffering in Uganda, thanking the Government of Uganda for their support over the years, along with all those who have helped to develop and shape palliative care in the country and ensuring that palliative care thrives in Uganda. She stressed the importance of inspiring young health professionals about palliative care as they are the future providers and leaders of palliative care in Uganda. Following on from this a dance was performed by ‘Dance with Valentino’ and the team from Hospice Africa Uganda depicting the 30-year journey of palliative care in Uganda.

The Keynote address was given by Dr. Meg O’Brian, Vice President of Global Cancer Treatment at the American Cancer Society (ACS). She discussed the prerequisites for scaling up the availability, accessibility, quality and equity to all in need, sharing lessons from the ACS Treat the Pain Initiative and the many years she has been coming to Uganda. She shared some of the key lessons learnt from the HIV/AIDS epidemic and the key foundations for quality and key interventions to establish before scaling up, stressing the importance of having the foundations in place that then make scaling up and quality successful. The conference was then officially opened by the Commissioner for Non-Communicable Diseases – Dr Oyoo Akiya Charles, on behalf of the Minister of Health. He reiterated the Government’s commitment to reducing the burden of cancer and expanding the provision of palliative care, acknowledging the WHA resolutions on cancer [15] and palliative care [14]. He called upon all stakeholders to work together, to unite in the fight against cancer and in the provision of palliative care – noting that *‘Together we are stronger’*. Finally, Prof. Anne Merriman spoke as she launched her autobiography ‘That is How the Light Got In’. She stressed that whilst the book talks about her life, all that has been achieved has been done so in collaboration and with the support of the MoH. She also said that her focus is on the future and not the past and called upon everyone for further action to strengthen both cancer and palliative care services in Uganda. A small delegation of colleagues and friends, led by Mr. Connor McCann who was representing the Irish Ambassador to Uganda, were then called upon to help launch the book.

## Conference summary

The conference was held at the Speke Resort Munyonyo in Kampala, and brought together close to 450 clinicians, academics, human rights advocates, lawyers, clergy, researchers, social workers, policy makers, MoH officials and donors, representing over 30 organisations, to share lessons and learn from each other. Participants attended from Uganda, Kenya, Rwanda, Ethiopia, Somalia, Eritrea, UK, Norway, the Netherlands, Ireland, Japan, India and the USA. All the sessions were interpreted into sign language and the conference was organised into six main tracks:

1. Innovations and new technologies
2. Education, advocacy, policy and law
3. Health promotion, prevention and early detection
4. Family and community involvement and empowerment
5. Clinical care and symptom management
6. Psychological, social and spiritual care.

Throughout the tracks there were also several integrated themes including paediatrics, vulnerable populations, service development and research. The scientific programme included 19 plenary presentations across 4 plenary sessions, 67 oral breakout presentations, 5 workshops and 47 poster presentations (Table 1). Presentations were given across the continuum of care, from prevention through to end-of-life care and into bereavement, across the age span, looking at quantitative and qualitative research as well as service provision, with an equal split between cancer and palliative care.

During the opening ceremony the USA organisation Global Partners in Care gave their biannual award for a successful partnership and this went to Hinds Hospice in the US and Hospice Africa Uganda. Later in the conference lifetime recognition awards were also given to the following: Dr Edward Katongole Mbidde, Mr. Aloysius Kisule, Dr Elizabeth Namukwaya, Ms. Rose Kiwanuka, Dr Kasule Stephen, Yumbe Regional Referral Hospital and Bombo Military Hospital. At the close of the conference awards were given for the best oral and poster abstracts – the best oral abstract went to Caroline Osoru from Arua Regional Referral Hospital for her abstract on ‘Factors affecting the turn up of patients for palliative care in Arua Regional Referral Hospital in Arua Central Division, Arua City’ and the best poster abstract went to Beatrice Rukundo from UCI for her abstract on ‘*Patients satisfaction with nursing care among paediatric cancer patients attending UCI-Mulago, Kampala, Uganda’.* All of the abstracts were published in the Abstract book which can be downloaded from the conference website.

Key themes were identified in each of the tracks, and in the plenary sessions. Within the plenary session on day one after the opening ceremony, one key area for development that was shared by all speakers was the participation of East African researchers, clinicians and scientists on the world stage in order to adequately represent the needs and priorities of their patient population and services. Various challenges were highlighted including the disproportionate mortality in the East-African population due to cancer, the lack of accessibility of cancer and palliative care in rural settings and the under-representing of the African patients in scientific research that is centered in the west. Dr. Museene Safinah from the Ministry of Education and Sports highlighted the numerous outcomes achieved by the different education programmes and shared that the future of palliative and cancer education must include the diversification of the programmes to a wide range of professionals, in order to provide quality care. Delegates were then encouraged by Grace Nayiga from the Uganda Network on Law, Ethics and HIV/AIDS (UGANET), to act as advocates for our service users, highlighting the many needs that patients and their families may experience and how social justice is part of holistic care delivery. She recommended participants to work with legal institutions and community institutions as we scale up cancer and palliative care services in order to empower patients, their families and health workers. Through collaborations with human rights lawyers, social justice in cancer and palliative care can be enhanced in Uganda the East African region.

Prof Suzanne Turner, from the University of Cambridge in the United Kingdom, brought a message of hope in the treatment of childhood cancers, in particular in Burkitt’s lymphoma. She highlighted the importance of global collaboration and shared areas of current research in the area which is improving our understanding of the biology and treatment of lymphomas in children around the world. Delegates were then reminded by Dr. Andrew Katumba from Makerere University that innovation is key to problem solving in low-resource settings. He highlighted possible applications of digital science and artificial intelligence in cancer screening and care, including diagnostic support and analysing patterns in electronic records. He noted that the recent global pandemic highlighted telehealth as an emerging technology allowing satellite clinics, remote patient monitoring and personalised care, with community health workers being crucial in harnessing the benefits of telehealth technologies. Whilst recognising some of the challenges such as the exchange of health information and the hosting of data, he encouraged participants to embrace technology and not be fearful of it. The theme of new technologies was developed further by Prof. Warren Edus Hootie who highlighted the importance of the analysis of cancer genomes as cancers cases in Uganda are projected to double in Uganda between 2020 and 2040. He discussed the Cancer Genome Atlas and its work to detect genomic and epigenomic abnormalities within tumours, but noted the lack of participation of scientists from Africa in this work and he encouraged us to get involved so that the genomics revolution will provide maximum benefit for patients with cancer across Africa.

In the afternoon of the first day of the conference a panel discussion was held on health promotion, prevention and early detection and family and community involvement and empowerment. The discussion was facilitated by Dr. Muwanga Moses who represented Dr. Rony Bahatungire, the Acting Commissioner Health Services in Charge of Clinical Services from the MoH. Five panelists took part in the discussion – Dr. Richard Kabanda (MoH), Dr. Hafisa Kasule (WHO), Gertrude Nakigudde (Uganda Women’s Cancer Support Organisation), Lacey Ahern (Global Partners in Care) and Dr. Emmanuel Luyirika (African Palliative Care Association). During the discussion the differences between health promotion, health education and preventative health were highlighted and how good communication is vital and each of these strategies can empower the patient and help in public health and disease prevention. Important lessons with regard to partnerships were also highlighted including the fact that partnerships need time and resources – they start small and can grow over time and funding must be driven by front line partners on the ground to allow flexibility.

Dr Kasule discussed the WHO’s public health approach to cancer and palliative care in the region and shared WHO’s conceptual model for palliative care development [16]. This is about having the right policies in order to build palliative care programmes and ensuring it allows the appropriate provision of pain medicines. Education of professionals and patients is a key area of focus and the integration of palliative care should be at all levels of care, including primary care and also at the policy level. Finally, Ms. Nakigudde reflected on the lack of psychosocial support when she faced her breast cancer diagnosis 21 years ago. Her experience has made her an advocate for cancer and palliative care access in her community and at the national level, advocating for the needs of the patients and community in accessing high-quality care, as well as to raise awareness for cancer and palliative care services in Uganda.

Day 2, the 15th September 2023 started with a plenary session addressing symptom management, psychosocial and spiritual support. This was a diverse and engaging session which was opened by Dr. Solomon Kibudde (UCI) discussing the current status of radiation oncology in Uganda and the challenges they have faced as well as the need for capacity building. 50%–60% of cancers require radiation treatment at some point and despite the fact that there are now three treatment units, huge demands are still placed on limited personnel. He shared how they had moved from zero to ten radiation oncologists, and plans for ongoing training and capacity building. Dr. Charles Akiya (MoH) presented the strategic plan to control cancer based on the disease burden and risk factors, starting with prevention and progressing through treatment, palliative care and survivorship. The plan includes new chapters on ‘Cancer Control in Children’ and ‘Cancer in Special Interest Groups’. Thereafter many presenters shared a focus on early detection and screening, especially of cervical cancer, Kaposi sarcoma and breast cancer. The panel discussed methods of increasing the accessibility and availability of these interventions, and the importance of such as Uganda faces a growing burden of cancer. A key area for development noted was creating multi-disciplinary buy in and involving all stakeholders when developing new strategies and techniques.

Dr. Benjamin Mwesige presented on behalf of Dr. Henry Ddungu (UCI) with regards to emerging trends in the holistic management of haematologic malignancies explaining how new agents are producing better outcomes for individuals with leukaemia, however many are not yet available in Uganda due to cost. It is hoped that they will be available soon through collaborative efforts with multi-national partners. Dr. Aggrey Semeere (Infectious Diseases Institute) presented about the work of the laboratory to create a point of care test for Kaposi Sarcoma, which is both specific and sensitive, and can be used by trained nurses and phlebotomists. Alongside this photo identification of Kaposi Sarcoma using AI and pattern recognition is now being developed for screening and referral. It was fascinating to hear of these innovative new technologies and how they may transform care provision in the future. Dr Naghib Bogere (UCI) went on to highlight how we are seeing an uneven distribution of breast cancer cases across districts and how the team at UCI are trying to address this disparity through mobile screening, along with advances in the treatment of breast cancer through surgery, radiation, chemotherapy and hormone therapy. He advocated for the provision of newer treatments, more personnel and more training in order to reduce these disparities further.

Prof. Francis Ssali (Joint Clinical Research Centre) pointed out that sickle cell disease is a much higher contributor to overall mortality in sub-Saharan Africa than originally thought with mortality peaking at 5 years and in young adults. While Dr. Ssali highlighted some uplifting developments in the treatment of sickle cell disease that are emerging, especially the accessibility of care for children in Uganda, he also advocated for the early involvement of palliative teams in the care of children with sickle cell disease, due to the profound physical and psychological symptoms they face. Being aware of this, Prof Julia Downing (International Children’s Palliative Care Network and Palliative care Education and Research Consortium) highlighted the importance of the psychosocial and spiritual domains of treatment when we treat children facing life-limiting conditions. She discussed the importance of hope, but also of acknowledging the reality of situations, and discussed that whilst psychosocial and spiritual care are connected, the integration of these are often unmet needs of children and their families, highlighting financial challenges in particular.

Over the course of the 2 days, five workshops were held exploring issues around: access to essential medicines for individuals with cancer and palliative care in Uganda; enhancing national palliative care data reporting and utilisations; research and education; and aging and ageism. A closed workshop was also held for heads of organisations addressing sustainable corporate governance practice in the post-COVID-19 Pandemic Era. The experience shared and lessons learnt from the workshops continued to show the need for more documentation of good practices and wide dissemination of findings. A wide-range of issues were discussed throughout the different tracks both in the oral and the poster presentations (Table 2) with participants having the opportunity to actively engage in discussions, learning from each other, and discussing crucial issues that impact on scaling up availability, accessibility, quality and equity.

The conference was closed with a short summary of the proceedings, the awarding of the oral and poster abstract prizes, and the recognition that it had been a successful conference, that there was a great feeling of collaboration, optimism, sharing and a commitment to working together to scale up availability, accessibility, quality and equity.

## Conclusion

Despite the challenges posed by the COVID-19 pandemic, it was evident from the conference that there was a sense of optimism and a commitment to the ongoing development of cancer and palliative care services within the country. Whilst challenges were recognised throughout the session there was also a recognition of all that has been achieved over the years within the field of cancer care and over the past 30 years since the introduction of palliative care in the country. This conference, the first face-to-face conference for a few years, was a great opportunity to reconnect and to share and learn from each other. As the conference came to a close over a cup of tea, participants shared with each other their thoughts on the conference, what they had learnt and the way ahead. It was an exciting conference to be part of and as Uganda moves forward in the development of Universal Health Coverage, and the implementation of both the cancer [14] and palliative care WHA resolutions [15] we do so in the knowledge that we have come through the pandemic together, we have shared together, we have supported each other, and we have shown that we are resilient and committed to provide cancer and palliative care for all those in need in Uganda.

## Conflict of interest

The authors declare that they have no conflict of interest.

## Funding

There was no funding for the preparation of the conference report.

## Figures and Tables

**Table 1.** Papers per track.

Track	Oral	Poster	Total
Innovations and new technologies	8	4	12
Education, advocacy, policy and the law	15	7	22
Health promotion, prevention and early detection	8	11	19
Family and community involvement and empowerment	14	4	18
Clinical care and symptom management	15	18	33
Psychological, social and spiritual care	7	3	10
Total	67	47	114

**Table 2.** Key themes from the conference.

Track	Key themes
1. Innovations and new technologies	Digital data is scalable, accessible, easier, faster and efficient in reporting. Digital data helps with the management of the daily workload and helps target care to the economically and socially disadvantaged. Health workers are always using digital technology e.g., mobile phones and so this can be developed further. There are however challenges to the use of new technologies, but this shouldn’t deter us from using them.
2. Education, advocacy, policy and the law	Importance of cancer care and palliative care education training, mentorship and support supervision. Capacity building for children’s palliative care need to be prioritised. How oncology nursing training has improved cancer care. Personal experience re survivorship and also stigma needs to be shared.
3. Health promotion, prevention and early detection	A country wide awareness campaign in needed for early detection and diagnosis is required. There is need to strengthen the current population based cancer registries and set up more registries in the sub-regions to improve cancer data quality. Increased tobacco use is a major risk factor for oesophageal cancer with 79% of patients with oesophageal cancer presenting with late stage disease, therefore urgent need for prevention and early detection. There is a need to determine the pattern of disease and risk factors across different regions to inform targeted cancer control strategies in Uganda.
4. Family and community involvement and empowerment	Training of Village Health Teams improves on their cancer knowledge and palliative care; therefore, they can improve the provision of cancer and palliative care services in the refugee settlements. Refugees with severe chronic illnesses face stigma from the community. Family meetings with patients and their family are very important in decision making. There are some children that face the challenges of taking care of their sick parents, so they need to be supported in this and to enable them to go to school etc. We need to expand health care services to rural areas in order to provide more treatment.
5. Clinical care and symptom management	More prospective studies should be conducted to better understand causes of early mortality and poor overall survival among the cancer patients especially patients with leukaemia. We need to build capacity through training more health care workers. There is a need for proper coordination or creating pathway between health care workers and family members on appropriate use of antibiotics. Review treatment protocol to provide targeted treatment as opposed to administering the same treatment protocols for different cancer types. Equip regional cancer centers with structured screening and treatment services.
6. Psychological, social and spiritual care	The high prevalence of depression, anxiety and burnout among palliative care providers, caregivers and patients. It is important to explore coping strategies – both for patients and health professionals. A balance is required for physical, social, emotional, environmental, spiritual, occupational and financial wellness. There is a need for mental health resources in the workplaces for cancer and palliative care providers. Tailored training in psychological support should be provided to health workers in an occupational setting. A focus is needed on support networks and reflection amongst staff with the provision of time and space to facilitate this.
